# Regulatory needs and activities to address the retinoid system in the context of endocrine disruption: The European viewpoint

**DOI:** 10.1016/j.reprotox.2020.03.002

**Published:** 2020-04

**Authors:** Elise Grignard, Helen Håkansson, Sharon Munn

**Affiliations:** a European Commission, Joint Research Centre (JRC), Italy; b Institute of Environmental Medicine (IMM), Karolinska Institutet, Sweden

**Keywords:** AOP, Adverse Outcome Pathway, CF, conceptual framework, DRP, detailed review paper, EATS, estrogen, androgen, thyroid, steroidogenesis, EC, European Commission, ECHA, European Chemicals Agency, EDTA AG, Endocrine Disrupters Testing and Assessment Advisory Group, EFSA, European Food Safety Authority, EU, European Union, GD, Guidance Document, IATA, Integrated Approaches to Testing and Assessment, IPCS, International Programme on Chemical Safety, MoA, Mode of Action, REACH, Registration, Evaluation, Authorisation and Restriction of Chemicals, TG, Test Guideline, UNEP, United Nations Environment Programme, US EPA, United States Environmental Protection Agency, Retinoic acid, Retinoic acid receptor families (RARs, RXRs), Vitamin A, Endocrine disrupters, Adverse Outcome Pathway (AOP), Regulatory toxicology

## Abstract

Endocrine disruption continues to be a matter of high concern, and a subject of intensive activities at the public, political, regulatory and academic levels. Currently, available regulatory test guidelines (TGs) relevant to the identification of endocrine disrupters are largely limited to estrogen, androgen, thyroid and steroidogenesis (EATS) pathways. Thus, there is an increasing interest and need to develop test methods, biomarkers, and Adverse Outcome Pathways (AOPs), for identification and evaluation of endocrine disrupters in addition to the EATS pathways. An activity focusing on the retinoid system has been jointly initiated by the Swedish Chemicals Agency and the European Commission. The retinoid system is involved in fundamental life processes and has been described, in previous work at the OECD, as a system susceptible to environmental endocrine disruption, the disruption of which could contribute to the increasing incidence of certain disorders in humans and wildlife populations.

## Highlights

• Endocrine disruption is a complex and important toxicological problem to consider.
• The retinoid system is evolutionary conserved and central for endocrine regulation.
• Regulatory tests are missing for assessing the retinoid system at any life-stage.
• Ongoing work by OECD member states will identify knowledge gaps and testing needs.

## 1 Endocrine disruption – subject of high concern

Endocrine disruption is a matter of high concern with respect to the safety assessment of manufactured chemicals, and has been the subject of intensive activities across the public, political, regulatory, and academic spheres at global level over a number of decades.

**Fig. 1:**
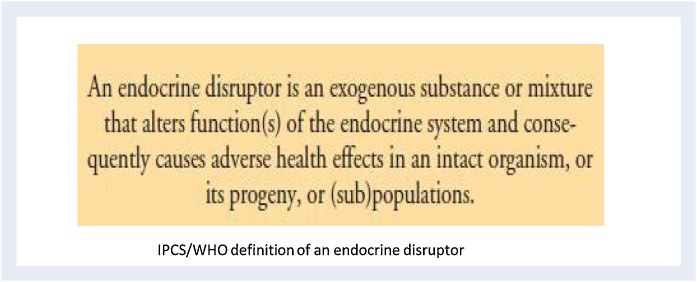
The IPCS/WHO definition of an endocrine disruptor (IPCS/WHO 2002).

Knowledge on endocrine disruption has increased immensely since the 1990´s as a result of many regulatory as well as research activities, including over 50 multinational collaborative research projects funded by the European Commission (EC) alone. Many of the research publications generated and other reports currently available demonstrate associations between exposures to various chemicals and human or environmental health effects, which can be linked to disruption of the endocrine system. Central reports include the IPCS Global Assessment of the State-of-the-Science of Endocrine Disruptors [1], its update, the UNEP/WHO expert report on the State of the Science of Endocrine Disrupting Chemicals – 2012 [2], the WHO report on Possible developmental early effects of endocrine disrupters on child health [3], and the EC report on the State of the art assessment of endocrine disrupters [4]. Included in the IPCS/WHO report of 2002 is the commonly agreed definition of a substance with endocrine disrupting properties (Fig. 1).

The Organisation for Economic Co-operation and Development (OECD) established an advisory group on the testing and assessment of endocrine disrupters (EDTA AG) already in the late 1990′s. A high-priority activity on endocrine disruption was initiated to revise existing test guidelines (TGs) with new endpoints and to enhance or develop new screening and testing methods with a focus on international coordination and harmonization of hazard characterization approaches. In the same period, the EC issued a Community Strategy for Endocrine Disrupters in 1999 (COM (1999) 706) [5]. To alleviate public concerns about endocrine disrupters the Community Strategy aimed to identify the problem, its causes and consequences, and to identify appropriate policy actions on the basis of the precautionary principle in order to respond quickly and effectively. The recent re-evaluation in November 2018 of the EC strategy resulted in an European Union (EU) framework on endocrine disruption (COM(2018)734) [6], which aims at minimising exposure to substances with endocrine disrupting properties, paying particular attention to important life stages, accelerating the development of research for effective and forward-looking decision-making, and promoting active dialogue with all stakeholders.

## 2 Current regulatory status for endocrine disrupters in Europe

**Fig. 2:**
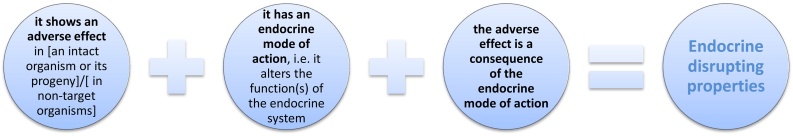
Scientific criteria for the determination of endocrine disrupting properties under the Biocidal (EU; 2017/2100) and Plant Protection Products (EU; 2018/605) Regulations as established by the European Commission. The criteria are based on the IPCS/WHO definition of an endocrine disruptor and state that “a substance shall be considered as having endocrine disrupting properties that may cause adverse effects in humans/or non-target organisms if, […], it is a substance that meets all of the following criteria, unless there is evidence demonstrating that the adverse effects identified are not relevant to humans/at the (sub)population level for non-target organisms. (1) it shows an adverse effect in an intact organism or its progeny / in non-target organisms, which is a change in the morphology, physiology, growth, development, reproduction or life span of an organism, system or (sub)population that results in an impairment of functional capacity, an impairment of the capacity to compensate for additional stress or an increase in susceptibility to other influences; (2) it has an endocrine mode of action, i.e. it alters the function(s) of the endocrine system; (3) the adverse effect is a consequence of the endocrine mode of action.

Within the framework of the Community Strategy, the EU has updated its body of laws to increase the protection of humans and the environment towards substances with endocrine disrupting properties. EU laws are currently among the most protective in the world and several pieces of European legislation related to risk management of marketed chemical substances, i.e. the legislation on chemicals in general (REACH), plant protection products, biocides, medical devices and water (water framework directive), contain specific provisions for endocrine disrupters (Table 1). Other legislation does not stipulate specific provisions, but instead subjects substances with endocrine disrupting properties to case-by-case regulatory action on the basis of the general requirements of the legislation. In September 2017, the EC published scientific criteria for the determination of endocrine disrupting properties for biocidal products (EU; 2017/2100 [7]), and in April 2018 for plant protection products (EU; 2018/605 [8]). The criteria are illustrated in (Fig. 2), and extension of these criteria to other pieces of legislation is under consideration.

**Table 1:** European legislation related to risk management of marketed chemical substances – Pieces of legislation, which contain specific provisions for endocrine disrupters.

Regulation	Reference	Comment
The REACH Regulation	EC No 1907/2006 [45]	*The following substances may be included in Annex XIV in accordance with the procedure laid down in Article 58:*
		*[…]substances — such as those having endocrine disrupting properties or those having persistent, bioaccumulative and toxic properties or very persistent and very bioaccumulative properties, which do not fulfil the criteria of points (d) or (e) — for which there is scientific evidence of probable serious effects to human health or the environment which give rise to an equivalent level of concern to those of other [CMR, PBT, vPvB] substances […] and which are identified on a case-by-case basis in accordance with the procedure set out in Article 59*.
The Biocidal Products Regulation	EU No 528/2012 [46]	*… the following active substances shall not be approved:*
		*(d) active substances which, […] are considered as having endocrine-disrupting properties that may cause adverse effects in humans or which are identified in accordance with Articles 57(f) and 59(1) of Regulation (EC) No 1907/2006 as having endocrine disrupting properties;*
The Regulation on Plant Protection Products	EC No 1107/2009 [47]	*An active substance, safener or synergist shall only be approved if, on the basis of the assessment of Community or internationally agreed test guidelines […], it is not considered to have endocrine disrupting properties that may cause adverse effect in humans/ non-target organisms, unless the exposure […] to that active substance, […] in a plant protection product, under realistic proposed conditions of use, is negligible*
The Regulation on Medical Devices	EU 2017/745 [48]	*Devices, or those parts thereof or those materials used therein that:*
		*— are invasive and come into direct contact with the human body,*
		*— (re)administer medicines, body liquids or other substances, including gases, to/from the body, or*
		*— transport or store such medicines, body fluids or substances, including gases, to be (re)administered to the body,*
		*shall only contain the following substances in a concentration that is above 0,1 % weight by weight (w/w) where justified pursuant to* Section 10*.4.2:*
		*[…]*
		*(b) substances having endocrine-disrupting properties for which there is scientific evidence of probable serious effects to human health and which are identified either in accordance with the procedure set out in Article 59 of Regulation (EC) No 1907/2006 of the European Parliament and of the Council (2) or, once a delegated act has been adopted by the Commission pursuant to the first subparagraph of Article 5(3) of Regulation (EU) No 528/2012 of the European Parliament and the Council (3), in accordance with the criteria that are relevant to human health amongst the criteria established therein.*
The Water Framework Directive	2000/60/EC [49]	*Indicative list of the main pollutants*
		*4. Substances and preparations, or the breakdown products of such, which have been proved to possess carcinogenic or mutagenic properties or properties which may affect steroidogenic, thyroid, reproduction or other endocrine- related functions in or* via *the aquatic environment.*

A Guidance Document (GD) to accompany the criteria jointly drafted by the European Chemicals Agency (ECHA) and the European Food Safety Authority (EFSA), supported by the Joint Research Centre (JRC), was issued in June 2018 [9] and targeted both applicants (pesticide and biocide manufacturers) and assessors from regulatory authorities to aid in the identification of endocrine disrupters. This EU GD describes how to gather, evaluate and consider all relevant information for the assessment, conduct of a mode of action (MoA) analysis, and application of a weight of evidence (WoE) approach. Regarding regulatory testing and identification of relevant endpoints for endocrine disruption, the EU GD mainly refers to the OECD GD 150 [10] on the evaluation of chemicals for endocrine disruption using standardised OECD TGs. Furthermore guidance is given on how evidence for “adversity” and “endocrine activity” can be obtained by applying the OECD Conceptual Framework (CF) for Testing and Assessment of Endocrine Disrupters. The GD 150 presents all the test methods available, under development or proposed, for the assessment of endocrine disrupters, advising on interpretation of test results from such standardised methods and recommending the potential next step in building evidence for evaluating chemicals in a regulatory context. Most of these tests, although not initially, developed for evaluation of endocrine disruption do include relevant endpoint(s). It includes the CF for Testing and Assessment of Endocrine Disrupters, which is organised around five levels of biological organisation and type of information provided (Table 2). In the 2018 revision of the GD 150, consideration of possible effects via the retinoic acid pathway, and apical responses to perturbations of the juvenile hormone and ecdysteroid modalities in arthropods were introduced. However, it was recognised that there is, as yet, no OECD TG with specific mechanistic endpoints allowing the identification of chemicals acting on these modalities. Importantly, the EU GD for the identification of endocrine disruptors states that when assessing evidence regarding the potential of a substance for endocrine disruption, all relevant information should be considered, i.e. evaluated evidence should not be restricted to data generated according to TGs.

**Table 2:** The OECD Conceptual Framework (CF) for Testing and Assessment of Endocrine Disrupters established and organised around five levels of biological organisation and type of information.

	Mammalian and Non Mammalian Toxicology
**Level 1** Existing Data and Non-Test Information	Physical & chemical properties, e.g., MW reactivity, volatility, biodegradability. All available (eco)toxicological data from standardized or non-standardized tests. Read across, chemical categories, QSARs and other *in silico* predictions, and ADME model predictions.
**Level 2** *In vitro* assays providing data about selected endocrine mechanism(s) / pathways(s) (Mammalian and non mammalian methods)	Estrogen or androgen receptor binding affinity (OECD TG 493). Estrogen receptor transactivation (OECD TG 455 & TG 457). Androgen transactivation assay (OECD TG 458). Steroidogenesis in vitro (OECD TG 456). MCF-7 cell proliferation assays (ER ant/agonist). Other assays as appropriate.

	Mammalian and Non Mammalian Toxicology	Mammalian and Non Mammalian Toxicology
**Level 3** *In vivo* assays providing data about selected endocrine mechanism(s) / pathway(s)	Uterotrophic assay (OECD TG 440). Hershberger assay (OECD TG 441).	Xenopus embryo thyroid signalling assay (When/if TG is available). Amphibian metamorphosis assay (OECD TG 231). Fish Reproductive Screening Assay (OECD TG 229). Fish Screening Assay (OECD TG 230). Androgenized female stickleback screen (OECD).
**Level 4** *In vivo* assays providing data on adverse effects on endocrine relevant endpoints	Repeated dose 28-day study (OECD TG 407). Repeated dose 90-day study (OECD TG 408). 1-generation reproduction toxicity study (OECD TG 415). Male pubertal assay (see GD 150, Chapter C4.3). Female pubertal assay (see GD 150, Chapter C4.4). Intact adult male endocrine screening assay (see GD 150, Chapter Annex 2.5). Prenatal developmental toxicity study (OECD TG 414). Chronic toxicity and carcinogenicity studies (OECD TG 451-3). Reproductive screening test (OECD TG 421). Combined 28-day/reproductive screening assay (OECD TG 422). Developmental neurotoxicity (OECD TG 426).	Fish sexual development test (OECD TG 234). Fish Reproduction Partial Lifecycle Test (when/If TG is Available). Larval Amphibian Growth & Development Assay (OECD TG 241). Avian Reproduction Assay (OECD TG 206). Mollusc Partial Lifecycle Assays (OECD TG 242 & TG 243). Chironomid Toxicity Test (TG 218 & TG 219). Daphnia Reproduction Test (with male induction) (OECD TG 211). Earthworm Reproduction Test (OECD TG 222). Enchytraeid Reproduction Test (OECD TG 220). Sediment Water Lumbriculus Toxicity Test Using Spiked Sediment (OECD TG 225). Predatory mite reproduction test in soil (OECD TG 226). Collembolan Reproduction Test in Soil (OECD TG 232).
**Level 5** *In vivo* assays providing more comprehensive data on adverse effects on endocrine relevant endpoints over more extensive parts of the life cycle of the organism.	Extended one-generation reproductive toxicity study (OECD TG 443). 2-Generation reproduction toxicity study (OECD TG 416).	FLCTT (Fish LifeCycle Toxicity Test) (when TG is available). Medaka Extended One Generation Reproduction Toxicity Study Test (OECD TG 240). Avian 2 generation reproductive toxicity assay (when TG is available). Mysid Life Cycle Toxicity Test (when TG is available). Copepod Reproduction and Development Test (when TG is available). Sediment Water Chironomid Life Cycle Toxicity Test (OECD TG 233); Mollusc Full Lifecycle Assays (when TG is available). Daphnia Multigeneration Assay (if TG is available).

https://www.oecd.org/env/ehs/testing/oecdworkrelatedtoendocrinedisrupters.htm.

The level 1 refers to existing data, and existing or new non-test information, including physico-chemical properties or in silico predictions. The level 2 presents *in vitro* assays providing data about selected endocrine mechanism(s)/pathway(s). Levels 3–5 list *in vivo* assays, covering both mammalian and non-mammalian toxicology. The tests in level 3 are designed to provide information about selected endocrine mechanism(s)/pathway, whereas level 4 and 5 tests provide data on adverse effects on endocrine-relevant endpoints.

## 3 Available tools to support regulation of endocrine disrupters

So far, the main scientific focus of the regulatory TGs relevant to the detection and evaluation of substances with endocrine disrupting properties has largely been limited to estrogenic, androgenic, thyroid, and steroidogenesis (EATS) modalities. The first TGs developed with the aim of studying effects on the endocrine system were *in vivo* assays providing data on selected mechanism(s)/pathway(s), i.e. the *Uterotrophic assay* (TG 440 [11]) endorsed by the OECD in 2007, as well as the *Hershberger assay* (TG 441 [12]), the *Amphibian Metamorphosis Assay* (TG 231 [13]), the *Fish Short Term Reproductive Assay* (TG 229 [14]) and the *21 day fish assay* (TG 230 [15]) endorsed in 2009. The endocrine modalities investigated by the Test Guidelines are listed in Table 3.

**Table 3:** Endocrine modalities investigated in the OECD Test Guidelines (based on OECD GD 150).

Assay	CF level	Investigated endpoints inform on modalities*						
		E	A	S	T	JH	Ec	R
OECD TG 455 (2016): PBTG for Stably Transfected Transactivation In Vitro Assays to Detect Estrogen Receptor Agonists and Antagonists	**2**	**X**						
OECD TG 456 (2011): H295R Steroidogenesis Assay	**2**			**X**				
OECD TG 458 (2016): Stably Transfected Human Androgen Receptor Transcriptional Activation Assay for Detection of Androgenic Agonist and Antagonist Activity of Chemicals	**2**		**X**					
OECD TG 493 (2015): PBTG for Human Recombinant Estrogen Receptor (hrER) In Vitro Assays to Detect Chemicals with ER Binding Affinity	**2**	**X**						
OECD TG 229 (2012): Fish Short Term Reproduction Assay	**3**	**X**	**X**	**X**				
OECD TG 230 (2009): 21-Day Fish Assay	**3**	**X**	**X**	**X**				
OECD TG 231 (2009): Amphibian Metamorphosis Assay	**3**				**X**			
OECD TG 248 (2019): Xenopus Eleutheroembryonic Thyroid Assay (XETA)	**3**				**X**			
OECD TG 440 (2007): Uterotrophic Bioassay in Rodents 3	**3**	**X**						
OECD TG 441 (2009): Hershberger Bioassay in Rats	**3**		**X**		**X**			
OECD TG 206 (1984): Avian Reproduction Test	**4**							
OECD TG 210 (2013): Fish Early Life Stage Toxicity Test	**4**				**X**			
OECD TG 211 (2012): Daphnia Reproduction Test (with male induction)	**4**					**X**	**X**	
OECD TG 218–219 (2004): Chironomid Toxicity Test	**4**					**X**	**X**	
OECD TG 234 (2011): Fish Sexual Development Test	**4**	**X**	**X**	**X**	**X**			
OECD TG 241 (2015): Larval Amphibian Growth and Development Assay	**4**	**X**	**X**	**X**	**X**			
OECD TG 242 (2016): Potamopyrgus antipodarum Reproduction Test	**4**							**X**
OECD TG 243 (2016): Lymnaea stagnalis Reproduction Test	**4**							**X**
OECD TG 407 (2008): Repeated Dose 28-Day Oral Toxicity Study in Rodents	**4**	**X**	**X**	**X**	**X**			**X**
OECD TG 408 (2018): Repeated Dose 90-Day Oral Toxicity Study	**4**	**X**	**X**	**X**	**X**			**X**
TG 409 (1998): Repeated Dose 90-Day Oral Toxicity Study in Non-Rodents	**4**	**X**	**X**	**X**	**X**			**X**
OECD TG 410 (1981): Repeated Dose Dermal Toxicity: 21/28-Day Study	**4**	**X**	**X**	**X**				**X**
OECD TG 411 (1981): Subchronic Dermal Toxicity: 90-Day Study	**4**	**X**	**X**	**X**	**X**			**X**
OECD TG 412 (2018): 28-Day (Subacute) Inhalation Toxicity Study	**4**	**X**	**X**	**X**	**X**			**X**
OECD TG 413 (2018): Subchronic Inhalation Toxicity: 90-Day Study	**4**	**X**	**X**	**X**	**X**			**X**
OECD TG 414 (2018): Prenatal Developmental Toxicity Study	**4**	**X**	**X**	**X**	**X**			**X**
OECD TG 421 and 422 (2016): (Combined Repeated Dose Toxicity Study with the) Reproduction/Developmental Toxicity Screening Test	**4**	**X**	**X**	**X**	**X**			**X**
OECD TG 426 (2007): Developmental Neurotoxicity Study	**4**	**X**	**X**	**X**	**X**			**X**
OECD TG 451-3 (2018): Combined Chronic Toxicity/Carcinogenicity Studies	**4**	**X**	**X**	**X**	**X**			**X**
OECD TG 233 (2010): Sediment Water Chironomid Life Cycle Toxicity Test	**5**					**X**	**X**	
OECD TG 240 (2015): Medaka Extended One-Generation Reproductive Toxicity Study	**5**	**X**	**X**	**X**	**X**			
OECD TG 416 (2001): Two-Generation Reproduction Toxicity Study	**5**	**X**	**X**	**X**	**X**			**X**
OECD TG 443 (2018): Extended One-Generation Reproductive Toxicity Study	**5**	**X**	**X**	**X**	**X**			**X**

Modality abbreviations: E; estrogen, A; androgen, T; thyroid, S; steroidogenesis, JH; juvenile hormone, Ec; ecdysone, R; retinoid -related modalities.

*some endpoints are only optional.

In parallel, the first *in vitro* test to study estrogenic potential of chemicals, i.e. the Stably Transfected Human Estrogen Receptor-α Transcriptional Activation Assay for Detection of Estrogenic Agonist-Activity of Chemicals (TG 455 [16]) was also adopted in 2009. More recently, some pre-existing TGs were updated to incorporate EATS-relevant endpoints such as the update in June 2018 of the TG 408 for a *Repeated dose 90-day oral toxicity study in rodents* [17] to include thyroid-related endpoints, and the update of TG 414 for the *Prenatal developmental toxicity study in rats* [18] to include measurements of anogenital distance in foetuses and thyroid hormones in dams. These recent updates highlight the fact that work is still ongoing in relation to further developing and updating existing TGs to be able to effectively identify substances acting via the EATS modalities. Another example of ongoing activities relates to efforts to combine the current *in vitro* test systems with metabolising ones, and thus take into consideration the potential impact of test-substance metabolites on the test result.

In parallel, it has been recognised that some TGs may also inform on other endocrine modalities, such as those related to the perturbations of juvenile hormone, ecdysone, and retinoic acid(s), although no TGs have specific mechanistic endpoints that would be diagnostic for these modalities. It should be noted that the different endpoints investigated in TGs provide different types of evidence. For example, direct evidence for adversity at the whole organism level can only be obtained from *in vivo* assays, i.e. levels 4–5 of the OECD CF, while evidence for endocrine activity can be obtained from *in vitro* and *in vivo* assays, as well as by using *in silico* tools to model the activity. One example of the latter is provided by the ToxCast Estrogen Receptor model, where results from *in vitro* tests are integrated into a computational model to conclude on estrogenic activity [19]. Some endpoints investigated *in vivo*, due to the nature of the effect and the existing knowledge, may also inform on a specific endocrine modality. For example, hypospadias accompanied by decreased anogenital distance and nipple retention in male rats would be indicative of anti-androgenic activity [20]. The OECD TGs which can be used to evaluate substances for their endocrine properties and the endocrine modalities investigated are presented in Table 3.

## 4 Endocrine disruption beyond EATS

The EDTA AG of the OECD published in 2012 a Detailed Review Paper (DRP 178 [21]) on the state of the science on novel *in vitro* and *in vivo* screening and testing methods and endpoints for evaluating endocrine disrupters, which evaluated opportunities for extending screening and testing strategies for endocrine disruption. Focus was on possibilities to develop test methods and molecular effect biomarkers for toxicity screening and monitoring studies, which also can provide MoA information for critical endpoints in existing TGs. The emphasis of DRP 178 is on pathways beyond EATS, for which both (i) significant evidence of susceptibility to disruption by environmental concentrations of chemicals with potential for adverse outcome exists and (ii) assays for the detection of endocrine disruption are sufficiently developed for protocol standardization and validation. A list of assays to be considered for identification of endocrine disrupters through perturbations of various signalling pathways is reported in Table 4.

**Table 4:** Endocrine pathways and related assays considered in the DRP 178.

Signalling pathway	Assays
HPA	• Glucocorticoid receptor assay (in vitro) • ACTH release (in vivo) • Adrenal steroid synthesis (in vitro, in vivo) • Stress response (in vivo)

HPG	• Assay development reauired

Somatotropic	• IGF-1 levels (in vivo) • Growth (in vivo)

Retinoid	• RXR reporter assay (in vitro) • RAR reporter assay (in vitro) • AhR reporter assay (in vitro) • Adipocyte differentiation (in vitro) • Lipid accumulation (in vivo) • Serum retinoid levels (in vivo)

HPT	• TR reporter (in vitro) • Cell proliferation (in vitro) • Thyroid peroxidase (in vitro) • Iodide uptake (in vitro)

Vitamin D	• Assay development required

PPAR	• Transactivation reporter (in vitro) • Adipocyte differentiation (in vitro) • Peroxisome proliferation (in vivo) • Lipid accumulation (in vivo)


The assays listed are at different levels of development.

(http://www.oecd.org/officialdocuments/publicdisplaydocumentpdf/?cote=env/jm/mono(2012)23&doclanguage=en).

The retinoic acid signalling pathway is one of seven pathways considered by the DRP 178 to be susceptible to environmental endocrine disruption and for which relevant endpoints could be measured either through development of new OECD TGs or by adaptation of existing TGs. This signalling pathway has a broad involvement in fundamental life processes, including important roles in regulating reproduction, embryofetal development, as well as lipid homeostasis in mammals. The DRP 178 also highlighted that the retinoic acid receptors, RARs and RXRs, can self-dimerise, or, in the case of RXRs, can dimerise with other nuclear receptors such as TR (thyroid hormone receptor), PPAR (peroxisome proliferator activated receptor), LXR (liver X receptor), FXR (farnesoid X receptor) or VDR (vitamin D receptor) resulting in hormonal cross-talk, which should be considered as a key element of concern when investigating endocrine disruption. RXRs also dimerize with the xenobiotic-sensing receptors CAR (constitutive androstane receptor) and PXR (pregnane X receptor). Current knowledge clearly illustrates that the retinoid system is indeed an endocrine system, with retinoic acids being the *in situ* synthesized receptor ligands with intracrine as well as hormonal activity [22]. Furthermore, there is a complex metabolic machinery to control the uptake, transformation, and overall distribution of dietary forms of vitamin A^2^, i.e. mainly retinyl esters and β-carotene, as well as to control the process of transforming dietary vitamin A sources into signalling retinoic acid molecules on spatial and temporal scales [24]. The metabolic vitamin A/retinoid-machinery strictly controls extra- and intra-cellular retinoic acid concentrations, spatial and temporal gradients of such concentrations over the life course, as well as the overall uptake, storage, and transport of the various chemical forms of vitamin A in and between cells and tissues in vertebrates [24,25]; as well as invertebrates [25]. On the transcriptional level, there is evidence for direct control of 27 genes [26] by a retinoic acid-liganded RAR-RXR heterodimer, while, for 100 additional genes, firm evidence is still lacking [26]. Furthermore, more than 100 genes seem to be indirectly regulated by retinoic acid complexes with RAR/RXR heterodimers, i.e. independently of a retinoic acid response element (RARE) on the DNA.

## 5 The way forward in identification and testing of retinoid disrupters

Adoption of a broader view on the endocrine system, as presented in DRP 178, is a major conceptual and scientific advancement for mechanistic as well as regulatory toxicology. The Adverse Outcome Pathway (AOP) framework (OECD GD 184 [27]) is another conceptual advancement to support the building and organisation of knowledge in endocrine toxicology and beyond (as also emphasized in DRP 178). An AOP is an analytical construct that describes a sequential chain of causally-linked events at different levels of biological organisation that can lead to an adverse health outcome or an ecotoxicological effect of regulatory relevance.

AOP(s) can be used to help identify substances, which act via an endocrine (retinoid) disrupting mode of action. They can be used within the regulatory context of endocrine disrupter identification by linking an adverse effect or outcome to a specific perturbation of the endocrine system.

AOP development can also help identify knowledge gaps, where more research is needed. An established AOP or AOP network can also be used to demonstrate mechanistic relevance of test methods to support their development towards regulatory use. Examples of such AOP networks related to neural tube and axial defects, and cleft palate, respectively in relation to retinoic acid signalling have been recently published [28,29]. These AOPs demonstrate on the molecular, cellular, tissue, and organ levels that inappropriate activation of RAR (mediating nuclear receptor response to retinoic acid), as well as inhibition of CYP 26 (involved in degrading retinoic acid) or Raldh (involved in synthesising retinoic acid from its precursor retinal) can alter the cellular metabolism and/or spatiotemporal distribution of retinoic acid, which in turn can result in irreversible skeletal effects, such as cleft palate, as well as cardiac or limb defects [28,29].

The early key events of an AOP, presenting evidence at the molecular or cellular level, are generally based on animal-free models, such as *in vitro* or *in silico* systems. The relationship between each key event (so-called response-response relationships), including the magnitude of change in one key event required to trigger the next key event, can inform on a potential adverse outcome. The ability to translate the perturbations at molecular and cellular level *in vitro* to the likely outcomes at the whole organism level still has many uncertainties. Nevertheless, as knowledge continues to grow and when a sufficient number of AOPs will be developed, it could be envisaged that adverse outcomes could be predicted, from knowledge of the endocrine pathway perturbed without the need of laboratory animal experiments, which is in accordance with the objectives of the European Directive 2010/63 on the protection of animals used for scientific purposes. The AOP-approach, thereby, provides an opportunity to follow the 3Rs principles (replacement, reduction and refinement) also for regulatory assessment of endocrine disruption. It is well established that the retinoid system is evolutionary conserved [25], and despite the existing large knowledge gaps, in particular for invertebrate species, it is clear that there are organizational and functional commonalities of the respective retinoid systems across taxa all the way to the last common ancestor of all bilaterians. In this context, it is likely that identification of conserved retinoid system-relevant key events in the AOP context has a large potential to help reduce animal testing needs by permitting extrapolation of knowledge across species.

In line with the OECD recognition of the need to broaden the scope of research and regulatory testing beyond the EATS modalities for endocrine disruption, the EC organised in 2015 an expert survey entitled "Identifying gaps in available test methods for evaluation of endocrine disrupters" [30] to gather input on key issues to be used as a basis for further discussions at a follow-up expert workshop in 2017 on “Setting priorities for further development and validation of test methods for evaluating endocrine disruption” [31]. Forty experts of fifteen countries, including experts from EFSA's Scientific Committee and Working Group on Endocrine Disruptors, ECHA's Endocrine Disruptor Expert Group and Risk Assessment Committee (RAC), European members of the Working Group of National Co-ordinators of the OECD TG programme (WNT), and additional experts in the field of endocrinology, were invited to participate to the survey. The experts were asked to rank endocrine-related diseases/disorders with respect to the need to develop new test methods to better identify the potential contribution of environmental chemicals to the occurrence of diseases/disorders, while considering the level of concern of these diseases/disorders such as severity of impact on life quality and rising prevalence in the population (Table 5A). It is interesting to note that the second and third ranked disorders involve non-EATS modalities, and more particularly that perturbation of retinoic acid signalling can be implicated in metabolic disorders (ranked second). The experts were also asked to rank the need for new test methods or new endpoints to be added to existing OECD TGs to cover additional pathways. The criteria considered to determine the priority on methods/endpoints were, in decreasing order: relevance to diseases/disorders and to life stages/exposure windows; species and molecular target; animal welfare; and cost. The replies of the experts regarding the retinoic acid signalling pathway are presented in Table 5B. During the follow-up workshop [31], attendees were asked to rank endocrine-related diseases/disorders of highest concern, considering their severity, linkage to an endocrine mode of action, incidence, cost, societal impact, impact on younger people, ecosystem health, biodiversity, cost in terms of ecosystem services (*e.g.* pollinators), food security and safety, and to propose, as well as to prioritise methods and endpoints for filling the testing gaps related to these diseases/disorders. The prioritisation was based on a tiered approach considering the regulatory relevance, such as mode of action and adversity, then the practical feasibility, cost, and animal welfare. The views of the experts at the workshop, which are summarised in Table 6, highlighted the retinoid system in relation to the highly prioritised disorders of metabolic dysfunction and skeletal malformations in humans, and to interference with growth and development in environmental species (Table 6A). Various retinoid-relevant *in vitro* assays were suggested for further development, while recognising that several have already been developed and used as part of the US EPA ToxCast programme, and some retinoid-relevant *in vivo* measurements were proposed (Table 6B).

**Table 5:** Results from the expert survey on (A) the identification of gaps in available test methods for evaluation of endocrine disrupters based on diseases/disorders of concern, and (B) proposals to fill testing and endpoint gaps with a focus on the Retinoid system [30].

5A. Ranking of disease/disorder with respect to the level of concern (e.g. severity of impact on life quality and rising prevalence in the population) in humans and the need to develop methods to predict their development	
Rank*	Disease/disorder
1	Thyroid-related/neurodevelopmental disorders
2	Metabolic disorders
3	Immune system related disorders

5B. Priority of novel test methods or new endpoints to existing tests in order to cover retinoic acid signalling pathways		
Rank	Test method/Endpoint	Comments
Average – high	RAR/RXR transactivation	
Average	Retinoid-relevant endpoints in *in vivo* TGs	
	IATA for retinoic acid axis	
	weight gain, adipose tissue mass, lipid accumulation, retinoid level measurements	not specific markers for RAR/RXR signalling
Low	EROD-activity *in vivo*	
	Cyp1A expression of mRNA or protein *in vivo*	
	AhR transactivation	

* Ranking is from the highest concern (1) to the lower (10) in the whole survey. Rank 1 is the area where methods should be developed with highest priority.

RAR: Retinoic acid receptor, RXR: retinoid X receptor, TG: Test Guideline, IATA: Integrated Approaches to Testing and Assessment, EROD: Ethoxyresorufin-O-deethylase, AhR: Aryl hydrocarbon receptor.

**Table 6:** Results of the EC workshop on Setting priorities for further development and validation of test methods and testing approaches for evaluating endocrine disruptors [31] with regard to the retinoid system in terms of (A) its involvement in diseases/disorders and (B) available test methods/endpoints.

6A. Priority of diseases, disorders and adverse outcomes related to endocrine disruption with highest concern and the recognized involvement of the retinoid system.			
Rank	Concern	Disease/disorder	Retinoid system involvement
High	Human Health	Neuro-development / Thyroid	
High		Female reproduction	
High		Metabolic dysfunction	Yes
Moderate		Malformations (such as craniofacial defects, cleft palate)	Yes

High	Environmental Health	Reproductive health	
High		Growth and Development	Yes

6B. Priority of novel test methods or new end-points to be added to existing tests in order to cover the retinoic acid signalling pathway	
	Test method/Endpoint
In vitro*	RAR/RXR transactivation
	Cyp 26 ativity
*In vivo*	serum and liver retinoic acid levels

* It should be noted that some *in vitro* retinoid signalling assays have already been developed and used to screen many chemicals as part of the US EPA ToxCast Programme (https://cfpub.epa.gov/si/si_public_record_report.cfm?dirEntryId=336407).

## 6 The retinoid system at the OECD level

An increasing number of reports and articles in the published literature illustrates the broad scope of retinoid system involvement in fundamental physiological processes and functions during embryofetal development and over the life course throughout invertebrate and vertebrate evolution (reviewed in [24,[32], [33], [34], [35], [36], [37]]). Moreover, the literature also indicates that various categories of chemicals, alone or in combination, can perturb the retinoid system in experimental whole animal and cell models at exposure doses, which are of relevance for human and wildlife exposure situations (reviewed in [21,[38], [39], [40], [41], [42], [43], [44]]). Many reports demonstrate, in multiple species, that concentrations of retinoids in tissues as well as circulatory levels are affected by various classes of chemicals, including organochlorine pesticides, dioxins, PCBs, brominated flame retardants and organometals. Other reports demonstrate that retinoid system perturbations can occur through different molecular events, and furthermore, can have functional impact on multiple organs [41,43]. For example, cleft palate can be induced by triazoles (antifungal organochlorine insecticides) through CYP 26-mediated RAR activation, while cleft palate induced by dioxin (environmental contaminant) is an AhR-mediated event, which also is likely to involve the retinoid system [29,42]. Likewise, developmental neurotoxicity can be induced by disruption of the retinoid system, e.g. by inhibition of CYP26 by triazoles, or histone deacetylase inhibition by valproic acid, in both cases leading to altered retinoic acid signalling [42]. Despite this growing body of evidence, there is still a lack of regulatory TGs, which specifically capture perturbations of the retinoid system with respect to endocrine disruption as well as in regulatory toxicology in general. Therefore, by building on priorities from the DRP 178, and the further growing knowledge base on the retinoid system, a project to analyse the needs and benefits of incorporating the retinoid system into the OECD TG programme was included on the work plan of the OECD in 2015.

The broad scope of the retinoid system involvement in fundamental life processes motivated the project to explore, in the existing literature, if and how diverse types of toxicities and health outcomes over the life course might be linked to retinoid system alterations. The purpose of the project is to map the existing knowledge in detail and to identify knowledge gaps, with the aim to provide a DRP on the retinoid system in biology and toxicology to be used as a starting point for the development of screening and testing methods in the OECD TG programme and to support the development of retinoid system-relevant AOPs. The retinoid system project was proposed and initiated by the Swedish Chemicals Agency and the European Commission (as co-leads).

Initially, the consultation of a broad representation of regulatory and academic scientists took place during 2016–2017 to detail the DRP scope and to develop a first draft organised around a thorough review of retinoid biology and biochemistry in general, as well as the evolutionary conservation of the retinoid system (genes and pathways) across phyla, and cross-species comparisons of retinoid function. The draft depicted opportunities for quantitative measurements of different chemical retinoid forms in different tissues that could then be broadly applied to new and existing TGs, and considered the role of the retinoid system in organ formation and function using knowledge derived from animal experimentation, human and wildlife epidemiology studies, and public health and disease studies. The first draft also described the need to gather evidence on the ability of chemicals to interact and impact upon the normal function of the retinoid system in various organs, bearing in mind the extensive involvement of the retinoid system in biological processes. Following this first project step, the draft-DRP is being further developed by the Swedish Chemicals Agency (KemI) and through specific contracts organised by the OECD and funded by the European Commission’s Directorate General for Environment. The final DRP will include chapters on male and female reproduction, neurodevelopmental disorders, as well as skeletal and craniofacial malformations. A Nordic Working Paper on the male and female reproduction will precede publication of the OECD DRP.

The regulatory interest in the DRP work on the retinoid system by OECD member countries encouraged the Elsevier journal of Reproductive Toxicology to initiate a Special Issue entitled *The Retinoid System in Biology and Toxicology*. Submissions were invited of original research, current reviews, commentaries, case reports and other information, in order to provide the general scientific community with up-to-date state-of-the-art information on the retinoid system in the regulatory context of endocrine disruption. It is expected that the combined publishing of the Nordic Working Paper, the OECD DRP, and the supporting special issue will stimulate a continued collaborative effort between the regulatory and academic sectors to address public and political concerns on chemical safety and the role the retinoid system may play in the complex nature of endocrine disruption.

## 7 Conclusion

The decision to investigate and analyse needs and benefits of incorporating the retinoid system into the OECD TG programme for endocrine disruption through a retinoid system DRP is building on priorities from the DRP 178, and is supported by OECD member countries. Important aspects behind the decision include the broad scope of retinoid system involvement in fundamental physiological processes and functions during embryofetal development and over the life course throughout invertebrate and vertebrate evolution, as well as the lack of current regulatory TGs, which specifically capture perturbations of the retinoid system in endocrine disruption.

The expected outcome of the retinoid system DRP, includes the identification of retinoid system-relevant key events to further develop the AOP framework, and the extension of existing AOPs and TGs incorporating aspects of the retinoid pathway. In turn it is expected that the retinoid system-relevant AOPs and TGs will help establish causal links between the molecular initiating events and the adverse outcomes; help develop regulatory relevant test methods and biomarkers; help reduce animal testing needs (3Rs principle) by permitting extrapolation across taxa; help identify regulatory-relevant gaps in retinoid system knowledge; and help focus available resources on the prioritised gaps.

## Declaration of Competing Interest

The authors declare that they have no known competing financial interests or personal relationships that could have appeared to influence the work reported in this paper.

## Footnotes

2 Vitamin A-active substances are compounds, which exhibit qualitatively the biological activity of retinol. The term retinoid on the other hand, includes both the natural and synthetic analogues of retinol with or without biological activity [23].
